# No gain in pain: psychological well-being, participation, and wages in the BHPS

**DOI:** 10.1007/s10198-020-01234-4

**Published:** 2020-09-22

**Authors:** Elena Lagomarsino, Alessandro Spiganti

**Affiliations:** 1 Department of Economics, University of Genoa, Genoa, Italy; 2 Department of Economics, European University Institute, Fiesole, Italy; 3 Department of Economics, Ca’ Foscari University of Venice, Venice, Italy

**Keywords:** Self-assessed mental health, GHQ, Sample selection, Endogeneity, Social support network, BHPS, I10, C23, H51

## Abstract

Accounting for endogeneity, unobserved heterogeneity, and sample selection in an unified framework, we investigate the effect of psychological well-being on wages and labour market participation using a panel from the British Household Panel Survey. We find the effect of psychological well-being on labour market outcomes to differ across gender. In particular, psychological distress significantly reduces participation across genders, but, conditional on participation, has a significant negative effect on hourly wages only in the female sample.

**Electronic supplementary material:**

The online version of this article (10.1007/s10198-020-01234-4) contains supplementary material, which is available to authorized users.

## Introduction

Mental health has been the target of global policy actions since the 2000s. Indeed, on top of the direct moral and social benefits, tackling mental ill health has considerable economic consequences. The costs of mental ill health include both direct costs for e.g. medication, visits to a clinic, and hospitalization, and large indirect costs for society through e.g. absenteeism, presenteeism (a situation in which individuals work at reduced productivity due to poor mental health), turnover, early retirement, and mortality. Recently, the spectrum of mental ill conditions considered by policy makers has widened to include milder forms (both diagnosed and undiagnosed), which are responsible for unpleasant emotional states and interfere with daily functioning, but do not affect insight or cognition. Indeed, the impact of these milder forms of mental ill health has become particularly relevant for labour market outcomes with the growth of the service industry and the transition towards knowledge- and emotion-intensive jobs, since they may lead to problems of concentration, loss of memory, decreased motivation, and distraction, thus reducing the emotional and cognitive resources needed to perform daily work tasks.

Thanks to a series of targeted strategies,^1^ the United Kingdom is among the most innovative OECD countries both in terms of awareness about the impact of mental health on employment outcomes and the level of integration of people with a mental illness into the labour force; nevertheless, it is also one of the countries bearing the highest direct and indirect costs of poor mental health [52]. For example, the Adult Psychiatric Morbidity Survey suggests that 17% of the UK adult population was experiencing at least a common mental disorder in 2014, with females more likely to be affected than males. “Thriving at work", an independent review of mental health and employers published in 2017, estimates the annual costs of poor mental health to the UK to be between $\pounds 79$ and $\pounds 99$ billion, including $\pounds 3$ billion of costs to the NHS, between $\pounds 37$ and $\pounds 52$ billion of lost output, and costs to employers and self-employers between $\pounds 33$ and $\pounds 44$ billion, including staff turnover, sickness absences, and presenteeism [65].

These reports provide some insights on the potential size of the indirect costs associated with mental ill health, but economic researches are needed to support these estimates by looking empirically for their significance and magnitude. Since Bartel and Taubman [5], a rich economic literature has estimated a negative correlation between diagnosed mental disorders and labour market outcomes; however, the economic literature focusing on milder forms of mental ill health is still in its early stage. Whereas major disorders have large effects on work capacity at the individual level, the greater prevalence of milder forms of mental ill health and psychological distress probably makes them the biggest overall burden for society; as a consequence, understanding this relationship seems necessary to fully outline the indirect public cost of mental ill health and to assess the outcomes of social policies carried out to improve people’s mental health. In this paper, we thus investigate the relationship between a self-assessed measure of psychological distress and average hourly wages (as a proxy for productivity). We exploit data drawn from the British Household Panel Survey, and consider males and females separately to account for the potential differences observed in the psychological and economic literature.

However, studying this relationship presents several challenges. First, causality can go in either direction: while psychological distress is likely to affect labour productivity and thus wages, one could also expect a reverse effect from employment to mental health. Second, unobserved individual factors, such as childhood circumstances and cognitive ability, could be correlated with both mental health status and labour market outcomes. Third, a problem of measurement error may arise, since we rely on a self-reported measure of mental health. Finally, non-random sample selection may result in inconsistent estimation of the effect of psychological well-being on income if one fails to take into account that labour market participation is itself influenced by psychological distress. To overcome these numerous challenges, we apply a series of different estimators to our longitudinal dataset; moreover, we account for correlated individual effects, endogeneity of the mental health variable, and sample selection in an unified framework, using the estimator proposed by Semykina and Wooldridge [62] and instrumenting mental health with the perceived social support network available to the individual.

Results show the signs of endogeneity for the mental health variable and that correcting for sample selection in the labour force is necessary at least for the female sample. Furthermore, we find that psychological distress significantly affects labour market participation across genders: individuals that may be suffering from psychological distress are between 6 and 20 percentage points less likely to be employed. Conversely, psychological distress has a significantly negative effect on wages only for the female sample: the wage gap between a non-distressed and a distressed female may be as high as $\pounds 1$ per hour. Even if more research is needed, we argue that these results are consistent with different reactions to psychological distress across gender: the reduction in the offered wage may reflect presenteeism for the female sample, whereas this may remain hidden behind increased absenteeism in the male sample.

This paper proceeds as follows. “Literature review” reviews the existing literature. “Econometric method and issues” presents our estimation procedures, while “Data” describes the dataset. “Results” shows the results. The conclusion is drawn in “Conclusions”.

## Literature review

Health status has been included as a determinant of earnings at least since Luft [39]. However, the pioneering study of the impact of mental health on labour market outcomes is due to Bartel and Taubman [5], who, using data on white male twins born between 1917 and 1927, found negative long-term effects of psychoses and neuroses on wages, employment, and hours worked. Since then, a rich body of literature has studied the relationship between diagnosed mental disorders and labour market participation, but only few papers have also considered the impact of mental health on wages, like we do.

Among these, Ettner et al. [21], Marcotte and Wilxox-Gok [41], and Chatterji et al. [13] all use data from the National Comorbidity Survey, a large-scale field survey of mental health in the United States. Ettner et al. [21] found that psychiatric disorders significantly reduce employment for both males and females, but the detrimental effect on income is found to be significant only for females when accounting for the likely endogeneity of mental health. Similarly, Marcotte and Wilxox-Gok [41] showed that diagnosed mental disorders negatively impacts on only women’s earnings. Chatterji et al. [13] found no effects of recent psychiatric disorder on earnings among employed individuals; however, the effect on labour force participation and employment is negative for both females and males.

More recently, Mitra and Jones [46] used data from the US National Survey of Alcohol, Drug and Mental Health Problems and found a positive correlation between developing a mental health condition and the probability of transitioning to non-employment, but no consistent effects on earnings. Hakulinen et al. [31, 32], using data for the whole Finnish population between 1988 and 2015, concluded that severe mental disorders are associated with significantly lower levels of employment, education, and annual income. Finally, the OECD [51, 53] has also been investigating the relationship between mental ill health and labour market outcomes, and generally found mental illnesses to be responsible for decreased labour supply, high rates of unemployment, and reduced productivity at work.

Whereas the studies above focused on diagnosed mental disorders, as far as we know only Contoyannis and Rice [16] and Peng et al. [56] studied undiagnosed mental illnesses in this context, like we do. The former found that worsening psychological health, measured using a reduced version of the self-assessed General Health Questionnaire, is associated with a decrease in hourly wages, but only for males; however, they did not study the impact of mental health status on labour market participation and employment. The latter captured the mental health status of the respondent with an index calculated on the frequency with which, over the 2 weeks before the interview, the respondent has been bothered by “having little interest or pleasure in doing things" and “feeling down, depressed, and hopeless", and found evidences that exhibiting these mild depressive symptoms reduces the likelihood of employment for both males and females, but that it does not significantly affect hourly wage. Furthermore, they examined the relationship between depressive symptoms and absenteeism, and observed that even a mild form of depression increases the number of sick days by 2 days per year for men, while they do not have any statistically significant effect on sickness absences for women.

Whereas both Contoyannis and Rice [16] and Peng et al. [56] tried to deal with the likely endogeneity of the mental health predictor and the unobserved heterogeneity, they did not consider the potential bias associated with self-selection. To correct simultaneously for all these econometric issues, in this paper, we use the estimator proposed by Semykina and Wooldridge [62], allowing us to study the effect of psychological well-being on both labour market participation and income. To our knowledge, this estimator has been only used in a similar context by Jäckle and Himmler [36] who, using data from the German Socio-Economic Panel, found a statistically significant effect of physical health on wages for males and a significant effect on labour market participation for females; however, they did not investigate the effect of mental health status.

## Econometric method and issues

The aim of this paper is to estimate the relationship between psychological well-being and wages for the entire sample, and not only for those individuals who are employed. Therefore, we formally specify our model as follows:

1a $$\begin{aligned} w^*_{it} =\,&\beta _0 + \varvec{x}_{it} \varvec{\beta }_1 +\varvec{y}^*_{it} \varvec{\beta }_2 +\alpha _i + \eta _{it} \end{aligned}$$

1b $$\begin{aligned} w^*_{it} =\,&w_{it}, \quad \varvec{y}^*_{it} = \varvec{y}_{it} \quad \text {if } S_{it}=1 \quad \text {and unobserved otherwise} \end{aligned}$$

1c $$\begin{aligned} S^*_{it} =\,&\gamma _0 + \varvec{z}_{it} \varvec{\gamma }_1 + k_i + e_{it} \quad \text {with } S_{it}=1 \text { if } S^*_{it}>0, \end{aligned}$$

where $w^*_{it}$ is a measure of hourly wage of individual $i=1,...,N$ at time $t=1,...,T_i$, $\varvec{\beta }_1$ is a vector of parameters associated with $\varvec{x}_{it}$ vectors of independent variables (including a measure of psychological well-being) that can be observed for all the individuals in the samples, and $\varvec{\beta }_2$ is a vector of parameters associated with $\varvec{y}^*_{it}$ vectors of variables that can be observed only if the individual works; $\alpha _i$ is a vector of unobservable time-invariant individual characteristics, whereas $\eta _{it}$ is a mean zero unobserved error term.

Equation (1a) is a Mincer’s [45] wage equation, modified to account for the impact of psychological well-being (among other variables) and for the panel structure of the dataset. As stated in (1b), we observe $w^*_{it}$ only if the individual participates to the workforce, $S_{it}=1$, where $S_{it}$ denotes market participation. Equation (1c) indicates that we observe participation only if the latent variable $S^*_{it}$, which represents the unobservable individual propensity to work, is positive. This depends on $\varvec{z}_{it}$, a superset of $\varvec{x}_{it}$.

As anticipated, the estimation of (1) involves a number of issues. First, due to the lack in the BHPS of an objective measure that can easily capture psychological well-being in quantitative terms, we need to resort to a self-assessed measure, which is likely to contain inaccuracies. Second, psychological distress can be correlated with unobserved characteristics that can also affect productivity and, hence, wages (e.g., genetic endowment). The consequence is a non-zero correlation between the health regressor and the error component. Third, the direction of the relationship between health and wages can be reversed: on one hand, if investment in health increases with salary, health should rise with wages [29]; on the other hand, there are many risk factors for mental health that may be present in the working environment, especially considering the pressures deriving from a dynamic and innovation-oriented economy [68]. This leads to a correlation between health and the period and individual specific error component of wages, $\eta _{it}$.^2^

Another potential issue is selection bias. Selection is not an issue if, for example, the decision to participate in the labour market is randomly determined. However, this is unlikely to be the case, as we expect some of the factors that determine participation to also influence psychological distress and income, such as low self-esteem, states of anxiety, and loss of focus. We will correct for sample selection below, but, for the time being, we start our analysis by disregarding the selection problem, and thus by estimating the wage equation in (1a) in isolation. Below is our estimation strategy.

We first estimate the wage equation in (1a) by Pooled OLS using cluster robust standard errors to account for cluster heteroskedasticity. If the regressors are not correlated with the errors, the estimator is unbiased, consistent, and efficient. Second, we implement a within (FE) estimator with cluster robust standard errors, thus assuming that the regressors are uncorrelated with the idiosyncratic error but without making any assumptions on the unobserved heterogeneity. The FE estimator, in fact, will be unbiased and consistent as *N* and/or *T* tend to infinity even if the regressors are correlated with $\alpha _i$.^3^

Third, we estimate the wage equation in (1a) through a two-stage least-squared (2SLS) and a within-2SLS (FE-2SLS) regression with cluster robust standard errors. We run these two instrumental variable approaches (IV) to deal with the endogeneity problem. IV requires the use of an instrument, i.e., a regressor that is predictive of the potentially endogenous mental status, but that is otherwise independent of the dependent variable of interest (here, the labour market outcomes). Following previous literature, we instrument psychological distress using a proxy for the perceived social support network of the individual (see “Instrument”). We test the validity of the instrument using Hansen–Sargan’s test of overidentifying restrictions.

At this point, it remains to deal with sample selection. Thus, in the last step, we run a regression based on Semykina and Wooldridge’s [62] estimator.^4^ To do so, we first follow Mundlak [50], Chamberlain [12], and Wooldridge [70], and explicitly model the correlation between the regressors and the unobserved heterogeneity. In particular, we write $k_i$ as a linear combination of a constant term, the group means of the time-varying regressors in $\varvec{z}_{it}$ (the so-called Mundlak effects), and a normally distributed error term $a_i$. Therefore, the participation equation in (1c) is updated to:

2 $$\begin{aligned} S^*_{it} = \gamma _0 + \varvec{z}_{it} \varvec{\gamma }_1 + \varvec{{\bar{z}}}_i \varvec{\theta } + \nu _{it}, \end{aligned}$$

where $\nu _{it} = e_{it} + a_i$ is an error term, independent from $\varvec{z}_{it}$ on which, as for $\eta _{it}$, no restrictions are imposed in terms of heteroskedasticity and serial correlation.

Two assumptions are required to implement the estimator proposed by Semykina and Wooldridge [62]. The first one is that $\eta _{it}$ is a linear function of $\nu _{it}$ and mean independent of $\bar{\varvec{z}}_{i}$ conditional on $\nu _{it}$. The second is that $\alpha _i$ is modelled as in Mundlak [50] and Chamberlain [12] as a sum of a constant, the group means, and an error $b_i$; since we think that our variables of interest, psychological well-being, may be correlated with $\eta _{it}$, as it stands the first assumption is not satisfied. For this reason, we remove the mental health variable from $\varvec{z}_{it}$, where it is substituted with the instrument, which is assumed to be uncorrelated with $\eta _{it}$. This new vector is called $\varvec{q}_{it}$; its time averages are indicated with $\bar{\varvec{q}}_{i}$.

Having done all these steps, the following equation is obtained:

3 $$\begin{aligned} w^*_{it} = \varphi _0 + \varvec{x}_{it} \varvec{\beta }_1 + \bar{\varvec{q}}_{i} \varvec{\varphi }_1 +\varvec{y}_{it}\varvec{\beta }_2 + \bar{\varvec{y}}_{i}\varvec{\varphi }_2 +\xi _t\lambda _{it} + r_{it}. \end{aligned}$$

In Eq. (3), we substituted $\bar{\varvec{x}}_{i}$ with $\bar{\varvec{q}}_{i}$ to satisfy Semykina and Wooldridge’s [62] requirement that $\eta _{it}$ be uncorrelated with $\bar{\varvec{z}}_{i}$. Moreover, $r_{it}$ is the sum of $b_i$ and $l_i$ where the latter is the remaining part of $\eta _{it}$ after including the Inverse Mills Ratios (IMRs), $\lambda _{it}$. These are obtained estimating *T* probit models of equation (2). As there is nothing preventing $\lambda _{it}$ to be correlated with $r_{is}$ for $s\ne t$, Eq. (3) can then be estimated using a Pooled 2SLS regression where $\bar{\varvec{q}}_{i}$, $\varvec{y}_{it}$, $\bar{\varvec{y}}_{it}$, and $\lambda _{it}$ are used as instruments. As suggested by Semykina and Wooldridge [62], we build standard errors robust to heteroskedasticity and serial correlation.

## Data

We construct an unbalanced panel using the available 238,996 observations from the British Household Panel Survey (BHPS).^5^ Unfortunately, questions regarding the respondent’s social support network (one of our instruments) are asked only once every 2 years: we thus limit our analysis to nine biennial waves covering the period between 1991 and 2007. We drop individuals who, in a given year, are self-employed, retired, still in education, in maternity leave, or attending a government training scheme. To allow for heterogeneity in coefficients across gender, we split the sample between men and women. In line with the State Pension age in the years covered, we only consider males aged 16–65 and females aged 16–60 at the time of the interview.

After excluding individuals who did not give valid answers for the variables used in the estimations, we obtain a sample of 62,686 observations (of which 31,413 males and 31,273 females), for a total of 7991 individuals who, on average, completed 5.04 waves. For those estimators which do not account for self-selection, we consider only those individuals in employment at the date of the interview. This restricted sample consists of 54,496 employees (26,670 males and 27,826 females).^6^

### Dependent variables

Since the BHPS does not provide a measure of hourly wage, we construct it as a weighted average of the gross usual monthly wage from first and second job. First, we obtain the hourly wage in the main job by dividing the usual gross monthly pay by the number of hours worked per month for the main work, including paid overtime. Analogously, we calculate the hourly wage for the individuals who have a secondary job. We then construct an overall average wage by taking a weighted average of the hourly wage in the main and the secondary jobs; the weights correspond to the proportions of total working time spent in each type of job. We find the average hourly wage to be higher for males ($\pounds 9.62$) than for females ($\pounds 7.63$).

Finally, we calculate the inverse hyperbolic sine (IHS) transformation of the wage. The IHS transformation precisely approximates the logarithmic one and has the advantage of being defined for zero and negative values. In this way, the regression estimates are improved: the outliers influence is damped down and, thus, heteroskedasticity is ameliorated (see Pence [55] and Georgarakos et al., [25] for more details).

Participation in the labour market is determined by the current employment status of the respondent.^7^

### Mental health variable

Our main independent variable is a measure of psychological distress, which we suppose endogenous. It consists of a reduced version of the General Health Questionnaire (GHQ), a self-administered psychometric screening tool originally used to screen for minor psychiatric disorders and now considered a robust indicator of subjective psychological distress [8, 15, 27, 28]. The version in the BHPS is the widely used GHQ-12, adopted also by the World Health Organization [26], which consists of 12 questions covering feelings of incompetence, anxiety, depression, difficulty in coping, and sleep disturbance, among others (see Online Appendix A.1). We consider this general measure of psychological distress preferable to specific conditions (like panic attack or depression), since it covers a wider range of aspects that are likely to influence labour market outcomes; moreover, it can provide a measure of severity which is often absent when relying on specific conditions.^8^

Indeed, by counting the number of questions to which the individual responds in the worse two categories (out of four), one obtains a 12-point “Caseness" score, which is widely used in medical, psychological, and sociological research and increasingly used in economics studies.^9^ We invert the Caseness score to obtain a measure ranging from 0 (the most distressed) to 12 (the least distressed). Similarly to Jäckle and Himmler [36], we apply the IHS transformation to this scale, $h_{it}$, so that our independent variable of interest is obtained as follows,^10^

$$\begin{aligned} \mathrm {Log Mental Health}_{it} = \ln \left( h_{it} + \sqrt{h_{it}^2+1} \right) . \end{aligned}$$

In the rest of the paper, we refer to this variable as psychological well-being. Its observed means are 3.00 for working males and 2.91 for working females. These values decrease to 2.67 and 2.45 for unemployed. Standard t tests confirm that the non-working groups have statistically lower means of this variable than the working groups, and thus are more distressed. Moreover, females report significantly worse psychological well-being than males.

To facilitate the comparison between results obtained from linear and non-liner models, we later report labour market outcomes using a relatively conservative cut-off of 4 in the Caseness score to divide the respondents into “normals" and “possible cases": indeed, the clinical literature using the GHQ-12 argues that the latter group has a significant higher probability of suffering from a psychological disturbance [e.g. 30, 63, 37, 47] and the NHS has recently used this same threshold to monitor the prevalence of probable mental ill health in England [48]. The percentages of “cases" are 10.5% and 16.5% for working males and females, respectively; these increase to 30.8% and 41.3% for unemployed.

### Instrument

To address the likely endogeneity of psychological well-being, we resort to the use of an instrument; in particular, we instrument psychological well-being with a measure of the perceived social capital of the individual.^11^ Indeed, since McKenzie et al. [44], a number of epidemiological studies have demonstrated a positive association between social capital and mental health [see 18, 67, forliteraturereviews]; moreover, social support variables have been used as instruments in the previous economics studies, where they were found to be significantly associated with better mental health status [1, 33, 54].^12^

Our measure of social capital is the perceived social support network of the individual, which we construct using the answers to the following questions: “Is there anyone who you:" (1) “can really count on to listen to you when you need to talk?", (2) “can really count on to help you out in a crisis?", (3) “can totally be yourself with?", (4) “feel really appreciates you as a person?", and (5) “can really count on to comfort you when you are very upset?". For each question, we assign 1 if the answer is positive, and 0 otherwise. Our instrument is obtained by summing up these responses.^13^

The resulting variable has an observed mean of 4.68 for working males and 4.83 for working females. These values decrease to 4.38 and 4.54 for unemployed respondents. Standard t tests confirm that females have a significantly stronger support network than males (also controlling for employment status), and that unemployed individuals have a significantly weaker support network than employed (also controlling for sex).

### Other regressors

The analysis includes also a full set of socio-demographic characteristics. We control for the marital status (the baseline category is married, having a civil partner, or living as couple) and for the number of dependent children in the household. Since children of different ages could have different effects on labour market outcomes, we consider separately the numbers of infants (aged 0–2 years), children (aged 3–11 years), and adolescents (aged 12–18 years) in the household. As an indicator of educational attainment, we include a dummy variable which indicates if the individual has a degree or a higher education attainment. We also include a dummy variable indicating if the individual is white. Finally, the model is completed with a vector of time dummies to control for inflation and aggregate productivity effects and a dummy to account for living in the capital.

An additional set of explanatory variables is used in the estimation of the wage equation. To capture the concavity of the earnings function, we include third degree polynomials of age and experience. We also include a vector of dummy variables indicating the occupational status of the individual, if employed in the private sector, and if she has undertaken any job-related training in the previous year. We account for the presence of an union at the individual’s workplace and for being a member of this union. Finally, we include the number of employees at the employee’s workplace.

The exclusion restrictions, i.e., those variable that drive participation but can be reasonably omitted from the wage equation, are: the IHS transformation of non-labour income, a dummy variable for having a partner, partner’s monthly gross pay, partner’s education, and third degree polynomials of the partner’s age and experience.

Variable definitions are in the Online Appendix Table A2. Summary statistics are presented in the Online Appendix Tables A3 and A4.

## Results

### Participation equation

The Online Appendix Tables A5 and A6 present the results for the participation equation, separately for men and women.^14^ The mental health predictor is statistically significant across all specifications: an improvement in the psychological well-being of the respondent is associated with a significant higher participation in the labour market. The coefficients of the IV regressions^15^ are larger than the corresponding ones from OLS and FE, indicating the presence of measurement errors in the mental health variable that bias the coefficients towards zero. Conversely, the correlation between the mental health variable and latent individual heterogeneity seems to be associated with an upward bias, as accounting for correlated individual effects (i.e., using fixed effects) reduces the magnitude of the mental health coefficient. The coefficient of interest remains strongly significant across genders even in the pooled probit models: the magnitude of the effect of mental health on labour participation decreases only when using the Semykina and Wooldridge’s [62] estimator that accounts simultaneously for endogeneity and correlated individual effects.^16^

To facilitate the comparisons of the results from linear and non-linear models, Table 1 presents participation probabilities of two average (male and female) individuals, who differs only with respect to their Caseness score. The male probit estimates show the probability difference between “normals" and “cases" to vary between 21.3 percentage point when the mental health is considered exogenous and 10.8 percentage points when mental health is considered endogenous. In the female samples, the probability difference varies between 6.6 percentage points when controlling for endogeneity and 14.9 when we do not. These findings are consistent with the previous literature: indeed, in terms of magnitude of the estimated coefficients, they fit in between the results found by researchers considering only more severe forms of mental ill health (like Ettner et al. [21], and Chatterji et al. [13] who estimated probability differences of approximately 14% for males and 13% for females), those considering diagnosed depression and anxiety disorders (Mitra and Jones [46], who estimated a significant point reduction in the probability of being employed between 6.5 and 7.3 percentage points, but do not split the sample by gender), and those by Peng et al. [56], who only considered symptoms associated with mild depression (and found probability differences of 4% for males and 2.3% for females).

**Table 1:** Participation probabilities (percent), males and females

		OLS	FE	2SLS	FE-2SLS	Naive Probit	Instr Probit
Males	Normals	87.2	85.9	90.0	87.4	86.0	84.4
	Cases	66.6	74.9	48.8	65.1	65.0	73.9
Females	Normals	91.4	89.9	95.7	91.8	90.2	88.6
	Cases	76.4	82.8	58.5	74.7	75.5	82.3

The Probit results include the Mundlak effects

There are several potential mechanisms that could explain lower participation probabilities for people suffering from psychological distress. First, distressed people might find it more difficult to commit to job hunting if they are demotivated, feeling down, or overwhelmed, or they might find the process more stressful; they may even be less aware of job opportunities if they are not part of a strong social network. Second, if psychological distress is associated with low self-esteem and confidence, they might refrain to apply for certain work positions; if it associated with anxiety and fear, they might not applying if they worry that a job may be too stressful or may lead to unfamiliar situations. Third, they might be inadvertently discriminated by potential employers during the application process: for example, interviewers often inquire about one’s ability to perform under demanding deadlines or in high-pressure situations, which might prove difficult when suffering from distress. Moreover, the fear of being stigmatised may lead to a refusal to volunteer personal information, which may leave a negative impression during an interview. Finally, distressed employees might be more likely to quit: this could be due, for example, to an unsupportive or openly hostile environment at the workplace, or with the difficulties associated with disclosing a state of psychological distress. Unfortunately, the information contained in the BHPS does not allow to investigate if one of these effects is prevalent or in which measure they coexist.

The effects of the other predictors are as expected. For both genders, age is significant, with the probability of entering the workforce increasing with age until it reaches a peak and begins to slowdown. The existence of another source of income significantly reduces the individuals’ labour market attachment for both sexes. The presence of children has an impact only on females participation, and it is found to be positive for children older than 2 years. While one could expect a negative effect on the participation of females of the presence of very young child, the positive effect here may be explained by the need to return to the workforce to provide additional economic support to the household. Having a first or higher degree increases the probability of participation, especially for females. Being white is associated with a significantly higher probability of being employed. The partner characteristics are generally not significant.

### Wage equation

In Tables 5 and 6, we present the results of our econometric estimations for the wage equation. The first two columns show results from the pooled OLS and FE estimations: the coefficient obtained using pooled OLS is non-smaller than the FE coefficient, suggesting a positive correlation between psychological well-being and latent individual heterogeneity. In the last three columns, we correct for the assumed endogeneity of the health variables using 2SLS, FE-2SLS, and the Semykina and Wooldridge’s [62] estimator, respectively. As explained above, we use the perceived social support network of the respondent as an instrument for the mental health variable; following Wooldridge [70], we also include as instruments the exclusion restrictions of the participation equation.

Before moving to the interpretation of the estimated coefficients, we briefly discuss the results of the tests on the validity of the instruments and the presence of selection bias. Table 2 summarizes the test statistics. We test the rank conditions using an F test on the joint significance of the instruments in the first stage of the 2SLS and FE-2SLS regressions; the null hypothesis of weak identification is rejected at any sensible level. Moreover, we strongly reject the hypothesis that the models are underidentified in both samples. Finally, we test for overidentifying restrictions using Hansen’s *J* statistics. For both samples, we strongly reject the null hypothesis that our instruments are valid in the 2SLS; in the FE-2SLS, conversely, the set of instruments appears appropriate, especially for the male sample.

**Table 2:** Tests statistics on the instruments, males and females

	Weak identification		Under identification		Over identification	
	2SLS	FE-2SLS	2SLS	FE-2SLS	2SLS	FE-2SLS
Males	15.66	19.68	148.03	155.75	88.5	61.36
			(0.00)	(0.00)	(0.00)	(0.60)
Females	14.77	12.42	79.54	51.77	8.33	19.34
			(0.00)	(0.00)	(0.00)	(0.04)

*P* values are in parenthesis. For weak identification, given the cluster robust standard errors, the relevant test statistic is the so-called “Kleibergen–Paap Wald rk F test"; critical values in this case are unknown, so we use Stock–Yoko critical values for the i.i.d. case

Semykina [61] argues that a rejection of the null hypothesis of valid instruments could be a signal of the presence of selection bias. As a further test of the presence of selection bias, Wooldridge [70] suggests to include the IMRs deriving from the *T* probit estimations of the participation equation as explanatory variables in the within regression and in the FE-2SLS, and to perform a Wald test on the joint significance of the IMRs coefficients. If the resulting statistic is greater than the critical value, one should reject the null hypothesis of no sample selection. As one can see in Table 3, we reject the null hypothesis in both 2SLS and FE-2SLS only for the female sample. We interpret this as suggesting that, while in the male sample, selection bias does not seem to be a problem, the female sample is not randomly selected, thus requiring the use of the procedure proposed by Semykina and Wooldridge [62].

**Table 3:** Tests on selection effects, males and females

	FE		FE-2SLS	
	Males	Females	Male	Females
Wald test	0.64	3.65	2.39	21.88
*P* values	0.7228	0.0006	0.9350	0.0027

Now looking at the estimated coefficients, we find a significant positive effect of psychological well-being on wages in most specifications for the female sample (with an elasticity between psychological well-being and hourly wage equal to 0.16 when correcting for all the econometric issues) but no significant effects in the male sample. To facilitate the comparison of the results from different models, Table 4 presents predicted hourly wages in GBP of two average (male and female) individuals, who differs only with respect to their “Caseness" score. A male in “normal" mental health is predicted to have an hourly wage that is between $\pounds 0.11$ and $\pounds 0.95$ higher than a “case". Accounting for correlated individual effects reduces the wage gap, whereas accounting for endogeneity increases it. For females, the wage gap is null when health is considered exogenous, but grows to $\pounds 1.50$ under the 2SLS estimator. Accounting for correlated individual effects shrinks the gap to $\pounds 0.37$, but when non random selection into the workforce is additionally considered the wage gap between a “normal" and a “case" female jumps to $\pounds 1.08$ (or, equivalently, 16%). These results are consistent with Ettner et al. [21] and Marcotte and Wilxox-Gok [41], who also found no significant effects for males but large and significant effects for females; our results are smaller in terms of magnitude, perhaps because they considered only more severe disorders (their estimated wage gaps for the female samples are approximately 29% and 49%, respectively, when accounting for endogeneity). Chatterji et al. [13] and Peng et al. [56] found no significant effects on wages.

**Table 4:** Hourly wage predictions (GBP), males and females

		OLS	FE	2SLS	FE-2SLS	SW
Males	Normals	8.31	8.07	7.94	8.39	7.88
	Cases	7.94	7.96	6.99	7.78	7.46
Females	Normals	6.42	6.45	6.66	6.45	6.58
	Cases	6.41	6.41	5.16	6.08	5.49

There are various possible interpretations of these results. One possible explanation is that employers discriminate females more than males with regard to psychological distress. Another potential explanation is that the losses in productivity are more apparent for women than for men, perhaps due to selection effects in different tasks or positions. Finally, a further interpretation may be connected to the concepts of presenteeism and absenteeism. Indeed, Ettner et al. [21] showed that mental disorders are negatively correlated with the number of hours worked only for males, and Peng et al. [56] found a positive and significant correlation between the number of work loss days and symptoms of mild depression only for the male sample. These evidences might suggest that women may tend to show up to work even when they are feeling mentally unwell whereas men may prefer to take a sick leave. If this was the case, the significant decrease in the offered wage in the female sample may reflect presenteeism, i.e., a situation of reduced functioning due, for example, to loss of focus or motivation, associated with lower productivity at work. Conversely, men’s reduced functioning due to increased psychological distress would lead to absenteeism, and thus only influence their actual number of working hours. Unfortunately, the BHPS does not contain exact information on working hours or on sick days, as it only reports the number of hours usually worked in a week.

We now turn our attention to the other independent variables (Tables 5, 6). For both samples, the coefficients on the polynomial of age and experience exhibit the expected signs, and are significant at 1% in all specifications. Education is significant and positive for females, indicating a rate of return on having a degree that ranges from 6% to 23%. For males, the coefficient is usually not significant. This might indicate that women tend to self-select in qualification types to show their abilities.

**Table 5:** Wage equation, males

	OLS	FE	2SLS	FE-2SLS	SW
Log mental health	0.006	0.006	0.171***	− 0.028	0.103
	(0.0083)	(0.0077)	(0.0608)	(0.0864)	(0.0879)
Age	0.110***	0.205***	0.116***	0.212***	0.181***
	(0.0105)	(0.0152)	(0.0105)	(0.0151)	(0.0144)
Age square	− 0.202***	− 0.346***	− 0.216***	− 0.385***	− 0.359***
	(0.0279)	(0.0373)	(0.0282)	(0.0365)	(0.0373)
Age cube	0.001***	0.002***	0.001***	0.003***	0.002***
	(0.0002)	(0.0003)	(0.0002)	(0.0003)	(0.0003)
Has a degree	0.204***	0.026	0.209***	0.014	− 0.017
	(0.0161)	(0.0477)	(0.0163)	(0.0498)	(0.0502)
Kids (0–2 years)	− 0.001	0.001	− 0.000	− 0.004	0.008
	(0.0118)	(0.0112)	(0.0123)	(0.0112)	(0.0117)
Kids (3–11 years)	0.017***	0.012**	0.020***	0.014**	0.016**
	(0.0056)	(0.0059)	(0.0059)	(0.0061)	(0.0065)
Teens (12–14 years)	− 0.004	− 0.000	− 0.005	− 0.000	0.004
	(0.0072)	(0.0076)	(0.0077)	(0.0080)	(0.0082)
London	0.162***	− 0.080*	0.189***	− 0.087*	− 0.077
	(0.0216)	(0.0453)	(0.0232)	(0.0526)	(0.0507)
White	0.048*		0.049*		
	(0.0247)		(0.0252)		
Widowed	0.091	0.112	0.094	0.113	0.119
	(0.0729)	(0.0769)	(0.0738)	(0.0724)	(0.0759)
Divorced or separated	− 0.076***	− 0.026	− 0.056***	− 0.026	0.007
	(0.0204)	(0.0228)	(0.0211)	(0.0247)	(0.0256)
Never married	− 0.088***	0.001	− 0.082***	− 0.006	0.015
	(0.0123)	(0.0169)	(0.0128)	(0.0175)	(0.0182)
Experience	0.009***	0.007***	0.011***	0.007***	0.011***
	(0.0025)	(0.0024)	(0.0025)	(0.0024)	(0.0025)
Experience square	− 0.069***	− 0.036*	− 0.085***	− 0.039**	− 0.080***
	(0.0193)	(0.0194)	(0.0199)	(0.0195)	(0.0195)
Experience cube	0.001***	0.001	0.002***	0.001	0.001***
	(0.0004)	(0.0004)	(0.0004)	(0.0004)	(0.0004)
Private sector	0.051***	0.004	0.051***	− 0.001	0.047***
	(0.0126)	(0.0180)	(0.0128)	(0.0183)	(0.0127)
Professional	0.485***	0.078***	0.482***	0.082***	0.459***
	(0.0218)	(0.0230)	(0.0221)	(0.0246)	(0.0221)
Manager	0.446***	0.075***	0.446***	0.073***	0.422***
	(0.0137)	(0.0162)	(0.0139)	(0.0170)	(0.0138)
Skilled non-manual	0.200***	0.009	0.201***	0.006	0.188***
	(0.0130)	(0.0170)	(0.0135)	(0.0175)	(0.0135)
Skilled manual	0.085***	− 0.008	0.085***	− 0.012	0.085***
	(0.0100)	(0.0109)	(0.0102)	(0.0118)	(0.0101)
Part-time job	− 0.685***	− 0.769***	− 0.615***	− 0.648***	− 0.635***
	(0.0492)	(0.0681)	(0.0481)	(0.0674)	(0.0485)
Number of employees	0.000***	0.000***	0.000***	0.000***	0.000***
	(0.0000)	(0.0000)	(0.0000)	(0.0000)	(0.0000)
Union at workplace	0.030***	0.037***	0.040***	0.034***	0.040***
	(0.0114)	(0.0121)	(0.0115)	(0.0127)	(0.0114)
Member of union	0.077***	0.043***	0.076***	0.046***	0.074***
	(0.0120)	(0.0142)	(0.0120)	(0.0150)	(0.0119)
Job training (lag)	0.045***	0.001	0.040***	0.000	0.035***
	(0.0078)	(0.0071)	(0.0080)	(0.0074)	(0.0079)
Constant	0.269**	− 1.217***	− 0.325		0.412
	(0.1309)	(0.2421)	(0.2338)		(0.4648)
Time (joint significance)	332.68***	2.35**	2,318.53***	19.95**	611.58***
*N*	26,194	26,194	23,268	20,695	21,530

Cluster robust standard errors are reported in parentheses

White is dropped in the SW column because of collinearity

* $$P<0.1$$, ** $$P<0.05$$, *** $$P<0.01$$

**Table 6:** Wage equation, females

	OLS	FE	2SLS	FE-2SLS	SW
Log mental health	0.015***	0.002	0.224***	0.093	0.160***
	(0.0055)	(0.0055)	(0.0384)	(0.0610)	(0.0619)
Age	0.133***	0.218***	0.135***	0.225***	0.167***
	(0.0104)	(0.0129)	(0.0110)	(0.0134)	(0.0136)
Age square	− 0.283***	− 0.434***	− 0.285***	− 0.436***	− 0.355***
	(0.0287)	(0.0329)	(0.0303)	(0.0340)	(0.0363)
Age cube	0.002***	0.003***	0.002***	0.003***	0.003***
	(0.0003)	(0.0003)	(0.0003)	(0.0003)	(0.0003)
Has a degree	0.230***	0.110***	0.226***	0.116***	0.059**
	((0.0147)	(0.0279)	(0.0147)	(0.0297)	(0.0293)
Kids (0–2 years)	0.093***	0.069***	0.097***	0.067***	0.090***
	(0.0118)	(0.0119)	(0.0129)	(0.0130)	(0.0136)
Kids (3–11 years)	− 0.019***	− 0.051***	− 0.020***	− 0.046***	− 0.028**
	(0.0057)	(0.0072)	(0.0061)	(0.0076)	(0.0753)
Teens (12–18 years)	− 0.037***	− 0.028***	− 0.032***	− 0.023***	− 0.017**
	(0.0061)	(0.0067)	(0.0067)	(0.0074)	(0.0075)
London	0.233***	0.099**	0.245***	0.085*	0.069
	(0.0177)	(0.0399)	(0.0184)	(0.0452)	(0.0468)
White	0.010		0.026		
	(0.0257)		(0.0258)		
Widowed	− 0.071**	− 0.002	− 0.029	0.030	0.067
	(0.0347)	(0.0427)	(0.0363)	(0.0471)	(0.0467)
Divorced or separated	− 0.009	0.012	0.019	0.014	0.029
	(0.0135)	(0.0167)	(0.0147)	(0.0186)	(0.0199)
Never married	− 0.016	− 0.042***	− 0.007	− 0.035**	− 0.025
	(0.0111)	(0.0143)	(0.0117)	(0.0153)	(0.0158)
Experience	0.015***	0.010***	0.016***	0.011***	0.015***
	(0.0026)	(0.0023)	(0.0028)	(0.0026)	(0.0027)
Experience square	− 0.104***	− 0.073***	− 0.108***	− 0.077***	− 0.102***
	(0.0257)	(0.0209)	(0.0281)	(0.0229)	(0.0274)
Experience cube	0.002***	0.001***	0.002***	0.001***	0.002***
	(0.0006)	(0.0005)	(0.0007)	(0.0005)	(0.0007)
Private sector	− 0.062***	− 0.016	− 0.064***	− 0.016	− 0.065***
	(0.0096)	(0.0130)	(0.0098)	(0.0136)	(0.0097)
Professional	0.518***	0.086***	0.517***	0.081***	0.486***
	(0.0292)	(0.0298)	(0.0296)	(0.0315)	(0.0292)
Manager	0.401***	0.099***	0.395***	0.085***	0.381***
	(0.0120)	(0.0152)	(0.0126)	(0.0161)	(0.0125)
Skilled non-manual	0.157***	0.021	0.159***	0.015	0.148***
	(0.0087)	(0.0134)	(0.0092)	(0.0145)	(0.0092)
Skilled manual	− 0.008	− 0.012	− 0.016	− 0.030*	− 0.018
	(0.0125)	(0.0145)	(0.0135)	(0.0163)	(0.0133)
Part-time job	− 0.137***	− 0.058***	− 0.138***	− 0.063***	− 0.144***
	(0.0096)	(0.0141)	(0.0101)	(0.0155)	(0.0101)
Number of employees	0.000***	0.000***	0.000***	0.000**	0.000***
	(0.0000)	(0.0000)	(0.0000)	(0.0000)	(0.0000)
Union at workplace	0.059***	0.046***	0.054***	0.052***	0.053***
	(0.0103)	(0.0108)	(0.0106)	(0.0116)	(0.0104)
Member of union	0.096***	0.052***	0.105***	0.052***	0.105***
	(0.0104)	(0.0130)	(0.0109)	(0.0143)	(0.0107)
Job training (lag)	0.042***	0.005	0.041***	0.003	0.038***
	(0.0066)	(0.0062)	(0.0072)	(0.0067)	(0.0070)
Constant	0.002	− 1.291***	− 0.684***		0.358
	(0.1251)	(0.1949)	(0.1752)		(0.3621)
Time (joint significance)	519.86***	5.34***	3,309.50***	31.42***	669.70***
*N*	27,363	27,363	24,223	21,550	22,976

Cluster robust standard errors are reported in parentheses

White is dropped in the SW column because of collinearity

* $$P<0.1$$, ** $$P<0.05$$, *** $$P<0.01$$

For both genders, there is a significant gradient in wages which reflects occupational status. Being a professional is associated with a slightly higher average hourly wage with respect to being a manager, but this could be an indicator that managers can also be compensated with perks which are not accounted for in the hourly wage. For women, being a manual skilled worker has a negligible effect on wage compared with being an unskilled worker, while the difference is more marked for men; this may be explained by the fact that blue collar work is done mostly by men in our samples. For men, working in the private sector increases hourly wage by up to 5% compared to those who work in the public sector. On the contrary, women who work in the private sector earn as much as 6.5% less than in the public sector. This may be due to weakest gender discrimination in the public sector if compared with the private one.

Men’s wages appear to be much more negatively affected by the choice of switching to part-time than women (but only 4% of the male sample is in part-time occupation, while the proportion increases to 34% for females). An increase in the number of employees at the individual’s work place is associated with a strongly significantly but negligibly rise in wages. Training is also associated with a significant increase in wages. As expected, unions have a positive impact on wages, and predictably, the effect is greater (almost double) for members than non-members still covered by union bargaining and renegotiation. In general, union membership appears to have a larger impact on wages for women than for men, which might be caused by positive selection of women in unions.

Having very young children significantly increases hourly wages for females. At least for the females, however, this result may be contaminated by the presence of simultaneity and endogeneity bias for which we do not correct for. The presence of children between 3 and 11 years has a positive and significant effect on the wage for men, while it impacts negatively the wage for females, as does the presence of teenagers in household. This may be due to lost opportunities of career advancements during maternal leave, when compared with women with no children, which do not seem to influence males. The marital status does not appear to be significant across genders.

## Conclusions

In this paper, we investigated the relationship between self-assessed psychological well-being and labour market outcomes. Unlike previous papers, we did not only focus on diagnosed mental disorders, but we considered the entire spectrum of unpleasant mental states that may cause psychological distress and result in reduced emotional and cognitive functioning. Furthermore, we addressed the numerous econometric issues that characterize this relationship (i.e., endogeneity, sample selection, and unobserved heterogeneity) in a comprehensive framework.

We provided evidences that correcting for non-random selection into the workforce is necessary at least for the female sub-sample. We showed that mental distress significantly decreases the probability of participating to the labour market, both for males and females. When investigating the direct effect of psychological well-being on hourly wages, we found it to have a significant and positive effect only for the female sample. As a consequence, our findings suggest that psychological distress is important both on the intensive and extensive margins for females, while it only influences males in their participation decision. Whereas this supports the idea of a negative correlation between psychological distress and wages at least for the female sample considered in this study, we caution the readers that claims about causal effect among these two variables may not be made; moreover, since our preferred specification involves the use of instrumental variables, it may not be appropriate to extrapolate our results to the wider population of workers.

It should be noted that the accuracy of the measure of psychological distress that we used in the paper is dependent on individuals providing reliable and accurate responses: indeed, self-assessed measures of health are prone to a potential positive bias, whereby respondents rationalise negative labour market outcomes by self-reporting low health. However, since the questions asked by the GHQ do not explicitly mention work ability, they should be less prone to this bias [38]. Conversely, if respondents have a perceived incentive to under-report mental illness, because of the fear of being stigmatised, socially sanctioned, or disgraced [7, 10], our results are likely to be a lower-bound of the true effect of psychological distress on labour market outcomes.

In conclusion, our results support the idea that even milder forms of mental ill health may have a significant impact on labour market outcomes, but this may differ across genders. Policymakers can try to reduce the associated indirect costs by funding assistance programs for those seeking job opportunities, designing health policies aimed at making psychological support easily accessible, and trying to raise employers awareness on the importance of creating a mentally healthy workplaces and promoting initiatives to help the staff managing stress. However, to inform employment policies, further research is needed on the mechanisms that induce presenteeism and on the productivity trade-off between impaired workplace performances and incentivised absenteeism, especially with a focus on the differences across gender.

## Electronic supplementary material

Below is the link to the electronic supplementary material.

Supplementary material 1 (pdf 139 KB)

## Footnotes

1 For example, the “Health, Work, and Well-being" initiative in 2005, the “Health and Social Care Act 2012", the “No Health Without Mental Health" strategy in 2012; the NHS “Mental Health Five Year Forward View" dashboard in 2016, and the “Every Mind Matters" platform in 2019.

2 Simultaneity can arise if wages influence health contemporaneously or with a lag in the presence of serial correlation in $\eta _{it}$.

3 We also implement a generalised least-squares regression, which we do not present, since the Sargan–Hansen statistic on the overidentifying restrictions indicates that the additional orthogonality conditions are not satisfied in both samples.

4 We choose this particular strategy, because, among the several estimators that one can use to address the problem of selection bias in a panel setting, the one proposed by Semykina and Wooldridge [62] not only allows for endogenous regressors but also does not require any known distribution of the errors in the equation of interest and the participation equation.

5 The data are provided by the UK Data Services and include information on the social, demographic, and economic characteristics of a representative sample of UK households. The BHPS started in 1991 with 5500 households and 10,300 individuals drawn from 250 areas of Great Britain. In 1999, additional samples of 1500 households in each of Scotland and Wales were added to the main sample; in 2001, an additional sample of 2000 households was added in Northern Ireland. In 2010, more than 80% of the BHPS participants were merged into Understanding Society, a new survey which unfortunately lacks some of our variables of interest (especially, those about the social support network of the respondent).

6 We refer the interested reader to the Online Appendix Table A1 for more details about the stepwise construction of our sample.

7 There are three measures of current employment status in the BHPS: that on the Household Composition Form; that arising from the direct status question; and that arising from the sequence of questions about whether the respondent did any paid work in the last week, whether away from a job, and whether seeking work. Since the first of these is likely to be reported by someone else, we ignore it. To select all those in employment, we use the last measure, as suggested by Taylor et al. [66]. The second measure, indeed, is a self-defined status: the inconsistencies in our panel, however, are small (approximately 350 observations) and the results are qualitatively the same.

8 An alternative measure of psychological distress that it is often used in empirical studies and clinical trials is the Mental Component Summary derived from the Short Form Survey; unfortunately, this is available for only one wave of the BHPS (the correlation coefficient between this variable and the Caseness score is 0.62 in our panel). The BHPS also sporadically provides a single-item life satisfaction question (“How dissatisfied or satisfied are you with your life overall?", which is measured on a scale from 1 to 7). We prefer the Caseness score for its multifacetedness, its focus on psychological well-being, and the larger sample size; nevertheless, the correlation coefficient between the two variables is 0.47 in our panel and the regression results maintain the same signs and significance levels as the one presented below (these are available on request).

9 For example, the GHQ-12 has been used to study the relationship between psychological distress and income inequality [69], financial behaviour [11], lottery wins [24], employment [23, 42], commuting [59], promotion [9], education [17], stock prices [57], and crime [20].

10 This choice is motivated by pragmatical reasons, as it allows us to use only one instrument when applying an instrumental variable approach and to maintain the non-linear structure suggested by earlier literature; moreover, it is defined for zero values:

11 Several variables have been used as instruments for mental health in this large literature, including parental history of mental health and substance problems [13, 21, 41, 49, 58], earlier onset of psychiatric disorders or substance use [2, 3, 13, 14, 21, 58], presence of physical chronic conditions [2, 40, 43], participation in physical activity [33], religiosity [1, 14, 34, 35, 40, 43], recent stressful events [3, 22, 33], state-level alcohol and illicit drug policies and prices [4, 19], and, similarly to this paper, the perceived social support network available to the respondent [1, 33, 54].

12 As noted in the previous studies [1, 14, 54], there are two limitations to this approach: that the identifying variable is a personal characteristic and that employment may be conveyed through an individual’s network. To address these reliability issues, we test below if the instrument is a reasonably strong predictor of psychological well-being and if it can be excluded from the labour market outcomes equation.

13 Following previous literature, we also investigated various measures of religiosity and volunteering activities, but did not find them predictive of mental health in our panel. We also investigated including in our measure of social capital a variable counting the number of organizations the respondent is an active member in (excluding unions). Moreover, we tried different functional forms of the included instrument. Since results are qualitatively and quantitatively similar to the one showed here, we decided to not include them: these, however, are available on request.

14 In this paper, we used Stata 15 by StataCorp [64] and the following user-written programs: Baum et al. [6] and Schaffer [60].

15 The equation is exactly identified; therefore, the Hansen *J* statistic is not available. We strongly reject the hypothesis of weak identification and that the models are underidentified in both samples.

16 Note that, while in the Semykina and Wooldridge’s [62] procedure used to estimate the wage equation, we perform different probit estimations, one for each year; here, we present the results for the entire period.
