# Supplementary material for: No gain in pain: psychological well-being, participation, and wages in the BHPS
Source: Eur J Health Econ. 2020 Sep 22;21(9):1375–89. doi: 10.1007/s10198-020-01234-4 (PMC7581575; doi:10.1007/s10198-020-01234-4)
Supplement: Supplementary file 1 — Supplementary material 1 (pdf 139 KB) [file 10198_2020_1234_MOESM1_ESM.pdf]

Online Appendix to “No Gain In Pain: Psychological Well-Being, Participation, and Wages in the BHPS”

Elena Lagomarsino* Alessandro Spiganti†

A Online Appendix

A.1 GHQ-12

The GHQ-12 consists of the following 12 questions: (i) “Have you recently been able to concentrate on whatever you’re doing?”, (ii) “Have you recently lost much sleep over worry?”, (iii) “Have you recently felt that you were playing a useful part in things?”, (iv) “Have you recently felt capable of making decisions about things?”, (v) “Have you recently felt constantly under strain?”, (vi) “Have you recently felt you couldn’t overcome your difficulties?”, (vii) “Have you recently been able to enjoy your normal day-to-day activities?”, (viii) “Have you recently been able to face up to problems?”, (ix) “Have you recently been feeling unhappy or depressed?”, (x) “Have you recently been losing confidence in yourself?”, (xi) “Have you recently been thinking of yourself as a worthless person?”, and (xii) “Have you recently been feeling reasonably happy, all things considered?”.

Half of the items in the GHQ are “positively” worded, the other half are “negatively” worded. The available responses to negative items are: “Not at all”, “No more than usual”, “Rather more than usual”, and “Much more than usual”; positive items have as possible responses: “Better than usual”, “Same as usual”, “Less than usual”, and “Much less than usual”. All items are rescored so that a low score is indicative of high psychological well-being, while higher scores indicate greater mental distress. The most common way to summarise these answers is by using a bimodal score: this is obtained by counting the number of questions to which the individual responds in the worse two categories, giving a 12 point “Caseness” score.

*Department of Economics, University of Genoa, Genoa, italy. Email: Elena.Lagomarsino@unige.it
†Department of Economics, Ca’ Foscari University of Venice, Venice, Italy. Email: Alessandro.Spiganti@unive.it.

A.2 Tables

Table A1: Stepwise adjustment of samples and average number of waves

	Males			Females			Total
	Obs.	Individ.	Waves	Obs.	Individ.	Waves	Obs.
Complete sample	110,221	15,399	11.71	128,775	16,981	12.05	238,996
Available social network info	50,776	13,405	5,71	60,779	15,357	5.87	111,555
Below retirement age	42,914	11,881	5.52	46,601	12,511	5.62	89,515
Above 16 yrs	42,729	11,828	5.53	46,423	12,457	5.62	89,152
Not retired	40,718	11,463	5.45	45,175	12,256	5.57	85,893
Not in education	37,847	10,521	5.46	41,625	11,093	5.57	79,472
Not in maternity leave	37,847	10,521	5.46	41,200	11,073	5.55	79,044
Not in education	37,847	10,521	5.46	41,625	11,093	5.57	79,475
Not in family care	32,305	9,676	5.14	32,113	9,746	4.98	64,418
Not in other labour force status	31,996	9,586	5.12	31,733	9,644	4.97	63,699
Valid info on other variables	31,413	9,430	5.11	31,273	9,529	4.96	62,686
Of which: Employed	26,670			27,826			54,496
Unemployed	4,743			3,447			8,190

Table A2: Variable Definitions

Variable	Label
IHS of Hourly Wage	ISH transformation of average hourly wage
Participation	1 if did paid work last week or if no work last week but has job
Log Mental Health	Log transformation of the inverted Caseness score
Social Support Network	Is there someone who will listen, help in a crisis, you can relax with, really appreciate you, you can count on to offer comfort
Age	Age at date of interview in years
Age square	Square of age at date of interview /100
Age cube	Cube of age at date of interview /100
Has a Degree	1 if 1st or higher degree
Kids (0-2 yrs)	Number of children in the household aged 0-2
Kids (3-11 yrs)	Number of children in the household aged 3-11
Teens (12-18 yrs)	Number of children in the household aged 12-18
London	1 if living in London
White	1 if white
Widowed	1 if widowed or survive civil partner
Divorced or Separated	1 if divorced, separated, or dissolved/separated from civil partner
Never Married	1 if never married
Experience	Spell in current job in years
Experience square	Square of spell in current job /100
Experience cube	Cube of spell in current job /100
Private Sector	1 if employed in Private Sector
Professional	1 if professional
Manager	1 if managerial
Skilled Non-Manual	1 if skilled non-manual
Skilled Manual	1 if skilled manual
Part-Time Job	1 if part-time
Number of Employees	Number of employees at workplace (max 1,000) as average of the categories (e.g. "1-2" is rescored as 1.5)
Union at Workplace	1 if covered non-member of union
Member of Union	1 if covered union member
Job Training (lag)	1 if received education or training related to current employment in the previous year
IHS of Non-Labour Income	ISH transformation of non-labour income
Partner's Monthly Pay	Spouse or partner's monthly gross pay
Has a Partner	1 if cohabits with lawful spouse or live-in partner
Partner's Age	Co-habitant partner's age at date of interview in years
Partner's Age square	Square of co-habitant partner's age at date of interview /100
Partner's Age cube	Cube of co-habitant partner's age at date of interview /100
Partner's Experience	Co-habitant partner's spell in current job in years
Partner's Experience square	Square of co-habitant partner's spell in current job /100
Partner's Experience cube	Cube of co-habitant partner's spell in current job /100
Partner Has Degree	1 if co-habitant partner has 1st or higher degree

Table A3: Summary Statistics, Males

	Count	Mean	Sd	Min	Max
IHS of Hourly Wage	26,268	2.773	.640	0	6.177
Participation	31,153	.850	.357	0	1
Log Mental Health	31,153	2.958	.507	0	3.180
Social Support Network	31,153	4.640	.974	0	5
Age	31,153	38.582	12.430	16	65
Age square	31,153	16.430	9.956	2.56	42.25
Age cube	31,153	756.130	649.896	40.96	2,746.25
Has a Degree	31,153	.152	.359	0	1
Kids (0-2 yrs)	31,153	.090	.307	-9	3
Kids (3-11 yrs)	31,153	.464	.826	-27	5
Teens (12-18 yrs)	31,153	.227	.550	-18	5
London	31,153	.065	.247	0	1
White	31,153	.961	.194	0	1
Widowed	31,153	.006	.078	0	1
Divorced or Separated	31,153	.055	.227	0	1
Never Married	31,153	.239	.426	0	1
Experience	26,516	4.901	6.552	0	50
Experience square	26,516	.669	1.714	0	25
Experience cube	26,516	13.526	53.926	0	1,250
Private Sector	26,268	.771	.420	0	1
Professional	26,268	.072	.259	0	1
Manager	26,268	.316	.465	0	1
Skilled Non-Manual	31,153	.114	.317	0	1
Skilled Manual	31,153	.252	.434	0	1
Part-Time Job	26,469	.044	.205	0	1
Number of Employees	26,388	250.076	329.024	1.5	1,000
Union at Workplace	26,253	.477	.499	0	1
Member of Union	26,253	.312	.463	0	1
Job Training (lag)	29,318	.301	.459	0	1
IHS of Non-Labour Income	31,153	2.719	2.742	0	10.857
Has a Partner	31,153	.226	.418	0	1
Partner's Monthly Pay	31,153	500.042	772.066	0	20,558.57
Partner's Age	31,153	10.134	20.751	0	98
Partner's Age square	31,153	5.333	13.188	0	96.04
Partner's Age cube	31,153	315.273	937.743	0	9,411.92
Partner's Experience	27,875	.603	2.718	0	46
Partner's Experience square	27,875	.077	.601	0	21.16
Partner's Experience cube	27,875	1.496	18.201	0	973.36
Partner Has a Degree	31,153	.025	.156	0	1

Statistics for partner's variables are conditional on non-missing data.

Table A4: Summary Statistics, Females

	Count	Mean	Sd	Min	Max
IHS of Hourly Wage	27,465	2.556	0.602	0	5.705
Participation	31,039	0.890	0.313	0	1
Log Mental Health	31,039	2.863	0.628	0	3.180
Social Support Network	31,039	4.802	0.722	0	5
Age	31,039	38.163	11.506	16	60
Age square	31,039	15.888	8.892	2.56	36
Age cube	31,039	707.524	554.982	40.96	2,160
Has a Degree	31,039	0.150	0.357	0	1
Kids (0-2 yrs)	31,039	0.060	0.247	0	2
Kids (3-11 yrs)	31,039	0.427	0.749	0	5
Teens (12-18 yrs)	31,039	0.262	0.566	0	4
London	31,039	0.068	0.251	0	1
White	31,039	0.964	0.185	0	1
Widowed	31,039	0.016	0.126	0	1
Divorced or Separated	31,039	0.105	0.306	0	1
Never Married	31,039	0.195	0.396	0	1
Experience	27,718	4.176	5.407	0	47
Experience square	27,718	0.467	1.172	0	22.09
Experience cube	27,718	7.711	30.784	0	1,038.23
Private Sector	27,465	0.574	0.494	0	1
Professional	27,465	0.030	0.171	0	1
Manager	27,465	0.324	0.468	0	1
Skilled Non-Manual	31,039	0.325	0.468	0	1
Skilled Manual	31,039	0.073	0.260	0	1
Part-Time Job	27,631	0.340	0.474	0	1
Number of Employees	27,568	215.448	321.698	1.5	1,000
Union at Workplace	27,436	0.518	0.500	0	1
Member of the Union	27,436	0.322	0.467	0	1
Job Training (lag)	30,028	0.325	0.468	0	1
IHS of Non-Labour Income	31,039	3.725	2.659	0	10.446
Has a Partner	31,039	0.232	0.422	0	1
Partner's Monthly Pay	31,039	939.236	1,284.774	0	31,333.33
Partner's Age	31,038	10.463	21.036	0	96
Partner's Age square	31,039	5.520	13.382	0	92.16
Partner's Age cube	31,038	326.550	951.369	0	8,847.36
Partner's Experience	27,674	0.641	2.787	0	43
Partner's Experience square	27,674	0.082	0.588	0	18.49
Partner's Experience cube	27,674	1.528	16.313	0	795.07
Partner Has a Degree	31,039	0.028	0.164	0	1

Statistics for partner's variables are conditional on non-missing data.

Table A5: Participation Equation, Males

	OLS	FE	2SLS	FE-2SLS	Naive probit	+ Mundlak	Probit	+ Mundlak
Log Mental Health	0.123***	0.059***	0.305***	0.069*	0.796***	0.577***		
	(0.0053)	(0.0052)	(0.0314)	(0.0360)	(0.0455)	(0.0540)		
Social Support Network							0.173***	0.053*
							(0.0207)	(0.0278)
Age	0.035***	0.025***	0.034***	0.026***	0.448***	0.347***	0.413***	0.304***
	(0.0062)	(0.0078)	(0.0063)	(0.0078)	(0.0555)	(0.0852)	(0.0547)	(0.0827)
Age square	-0.067***	-0.063***	-0.061***	-0.063***	-0.943***	-0.572**	-0.877***	-0.487**
	(0.0163)	(0.0181)	(0.0165)	(0.0181)	(0.1438)	(0.2223)	(0.1421)	(0.2156)
Age cube	0.000***	0.000**	0.000**	0.000**	0.006***	0.003	0.006***	0.002
	(0.0001)	(0.0001)	(0.0001)	(0.0001)	(0.0012)	(0.0018)	(0.0012)	(0.0018)
Has a Degree	0.112***	-0.015	0.114***	-0.015	1.235***	-0.474	1.193***	-0.403
	(0.0062)	(0.0213)	(0.0067)	(0.0212)	(0.1108)	(0.4016)	(0.1103)	(0.4217)
Kids (0-2 yrs)	0.001	-0.003	-0.001	-0.003	0.003	-0.025	0.013	-0.038
	(0.0065)	(0.0053)	(0.0069)	(0.0054)	(0.0818)	(0.0948)	(0.0771)	(0.0900)
Kids (3-11 yrs)	-0.010***	0.000	-0.011***	-0.000	-0.049	0.024	-0.035	0.035
	(0.0032)	(0.0029)	(0.0033)	(0.0030)	(0.0375)	(0.0544)	(0.0366)	(0.0519)
Teens (12-18 yrs)	-0.009**	-0.002	-0.007	-0.002	-0.071	-0.002	-0.079*	-0.017
	(0.0043)	(0.0038)	(0.0045)	(0.0038)	(0.0453)	(0.0624)	(0.0449)	(0.0601)
London	0.008	0.006	0.013	0.006	-0.077	0.100	-0.075	0.074
	(0.0123)	(0.0211)	(0.0127)	(0.0212)	(0.1274)	(0.3171)	(0.1272)	(0.3209)
Widowed	0.041	0.091***	0.051	0.092***	0.370	0.781**	0.263	0.667**
	(0.0402)	(0.0309)	(0.0396)	(0.0309)	(0.2254)	(0.3087)	(0.2220)	(0.3017)
Divorced or Separated	-0.066***	0.013	-0.045***	0.014	-0.275**	0.247	-0.335***	0.144
	(0.0137)	(0.0117)	(0.0143)	(0.0123)	(0.1075)	(0.1632)	(0.1049)	(0.1568)
Never Married	-0.047***	-0.013	-0.045***	-0.013	-0.429***	-0.202	-0.406***	-0.223
	(0.0096)	(0.0096)	(0.0098)	(0.0096)	(0.0887)	(0.1464)	(0.0904)	(0.1508)
White	0.048***		0.042***		0.568***	0.582***	0.591***	0.630***
	(0.0137)		(0.0139)		(0.1297)	(0.1544)	(0.1298)	(0.1535)

Table A5 Continued: Participation Equation, Males

	OLS	FE	2SLS	FE-2SLS	Naive probit	+ Mundlak	Probit	+ Mundlak
IHS of Non-Labour Income	-0.063***	-0.036***	-0.058***	-0.036***	-0.500***	-0.395***	-0.505***	-0.394***
	(0.0013)	(0.0014)	(0.0015)	(0.0014)	(0.0168)	(0.0177)	(0.0164)	(0.0172)
Has a Partner	0.056	0.013	0.081	0.014	-0.221	-0.469	-0.445	-0.756
	(0.0829)	(0.0661)	(0.0885)	(0.0660)	(1.3557)	(1.4260)	(1.2658)	(1.3481)
Partner's Monthly Pay	0.000***	0.000	0.000***	0.000	0.001***	0.000***	0.001***	0.000***
	(0.0000)	(0.0000)	(0.0000)	(0.0000)	(0.0001)	(0.0001)	(0.0001)	(0.0001)
Partner's Age	-0.001	-0.000	-0.003	-0.000	0.054	0.058	0.075	0.082
	(0.0068)	(0.0053)	(0.0072)	(0.0053)	(0.1080)	(0.1132)	(0.0999)	(0.1065)
Partner's Age square	0.003	-0.001	0.009	-0.001	-0.184	-0.218	-0.246	-0.285
	(0.0170)	(0.0131)	(0.0181)	(0.0131)	(0.2669)	(0.2779)	(0.2464)	(0.2613)
Partner's Age cube	-0.000	0.000	-0.000	0.000	0.002	0.002	0.003	0.003
	(0.0001)	(0.0001)	(0.0001)	(0.0001)	(0.0021)	(0.0021)	(0.0019)	(0.0020)
Partner's Experience	-0.002	-0.002	-0.002	-0.002	-0.041	-0.019	-0.043	-0.030
	(0.0040)	(0.0027)	(0.0041)	(0.0027)	(0.0470)	(0.0505)	(0.0456)	(0.0484)
Partner's Experience square	0.021	0.006	0.022	0.006	0.369	0.123	0.321	0.146
	(0.0398)	(0.0228)	(0.0389)	(0.0227)	(0.3878)	(0.4001)	(0.3779)	(0.3851)
Partner's Experience cube	-0.000	0.000	-0.001	0.000	-0.008	-0.002	-0.007	-0.002
	(0.0009)	(0.0005)	(0.0009)	(0.0005)	(0.0071)	(0.0070)	(0.0070)	(0.0067)
Partner Has a Degree	0.008	0.002	0.005	0.002	0.199	0.131	0.197	0.106
	(0.0124)	(0.0098)	(0.0129)	(0.0098)	(0.1717)	(0.1908)	(0.1644)	(0.1828)
Constant	0.068	0.477***	-0.486***		-5.710***	-9.097***	-3.696***	-6.443***
	(0.0792)	(0.1302)	(0.1216)		(0.7018)	(1.1055)	(0.6787)	(1.0899)
Time (joint significance)	10.23***	3.80***	61.21***	26.62***	22.11***	5.55	18.32**	5.63
Mundlak (joint significance)						411.46***		405.55***
N	27,875	27,875	27,875	24,890	27,875	27,875	27,875	27,875

Cluster robust standard errors are reported in parentheses; * $p < 0.1$, ** $p < 0.05$, *** $p < 0.01$

Table A6: Participation Equation, Females

	OLS	FE	2SLS	FE-2SLS	Naive probit	+ Mundlak	Probit	+ Mundlak
Log Mental Health	0.092***	0.039***	0.272***	0.062*	0.568***	0.362***		
	(0.0045)	(0.0038)	(0.0250)	(0.0321)	(0.0296)	(0.0346)		
Social Support Network							0.220***	0.052*
							(0.0234)	(0.0288)
Age	0.097***	0.050***	0.094***	0.051***	0.963***	0.706***	0.944***	0.707***
	(0.0072)	(0.0089)	(0.0075)	(0.0089)	(0.0732)	(0.1031)	(0.0732)	(0.1021)
Age square	-0.228***	-0.120***	-0.216***	-0.120***	-2.276***	-1.600***	-2.238***	-1.611***
	(0.0196)	(0.0221)	(0.0203)	(0.0220)	(0.1958)	(0.2748)	(0.1956)	(0.2712)
Age cube	0.002***	0.001***	0.002***	0.001***	0.017***	0.011***	0.016***	0.011***
	(0.0002)	(0.0002)	(0.0002)	(0.0002)	(0.0017)	(0.0023)	(0.0017)	(0.0023)
Has a Degree	0.056***	0.031**	0.053***	0.032**	0.820***	0.553*	0.817***	0.505*
	(0.0057)	(0.0147)	(0.0063)	(0.0149)	(0.1017)	(0.3058)	(0.1015)	(0.2870)
Kids (0-2 yrs)	-0.013	0.001	-0.012	0.002	-0.115	-0.058	-0.121	-0.063
	(0.0080)	(0.0066)	(0.0084)	(0.0067)	(0.0885)	(0.1067)	(0.0878)	(0.1055)
Kids (3-11 yrs)	0.054***	0.027***	0.045***	0.026***	0.290***	0.304***	0.323***	0.318***
	(0.0038)	(0.0036)	(0.0041)	(0.0037)	(0.0399)	(0.0561)	(0.0396)	(0.0553)
Teens (12-18 yrs)	0.060***	0.032***	0.058***	0.032***	0.349***	0.337***	0.345***	0.326***
	(0.0045)	(0.0040)	(0.0047)	(0.0040)	(0.0443)	(0.0589)	(0.0433)	(0.0574)
London	0.004	0.009	0.002	0.009	0.052	0.227	0.046	0.233
	(0.0100)	(0.0225)	(0.0104)	(0.0224)	(0.1246)	(0.3466)	(0.1244)	(0.3537)
Widowed	0.140***	0.018	0.160***	0.026	0.679***	0.153	0.549***	-0.047
	(0.0228)	(0.0256)	(0.0232)	(0.0283)	(0.1898)	(0.3113)	(0.1883)	(0.3011)
Divorced or Separated	-0.019*	-0.000	0.008	0.003	-0.089	-0.030	-0.172**	-0.117
	(0.0112)	(0.0100)	(0.0118)	(0.0108)	(0.0868)	(0.1268)	(0.0871)	(0.1267)
Never Married	-0.035***	-0.004	-0.032***	-0.003	-0.307***	-0.171	-0.302***	-0.187
	(0.0083)	(0.0089)	(0.0084)	(0.0092)	(0.0875)	(0.1338)	(0.0881)	(0.1343)
White	0.040***		0.040***		0.624***	0.704***	0.587***	0.647***
	(0.0145)		(0.0149)		(0.1462)	(0.1650)	(0.1479)	(0.1666)

Table A6 Continued: Participation Equation, Females

	OLS	FE	2SLS	FE-2SLS	Naive probit	+ Mundlak	Probit	+ Mundlak
IHS of Non-Labour Income	-0.049***	-0.026***	-0.044***	-0.026***	-0.416***	-0.308***	-0.422***	-0.309***
	(0.0014)	(0.0014)	(0.0015)	(0.0014)	(0.0169)	(0.0181)	(0.0169)	(0.0182)
Has a Partner	-0.033	0.035	-0.029	0.032	-0.342	0.396	-0.133	0.476
	(0.0759)	(0.0563)	(0.0841)	(0.0574)	(1.0566)	(1.1387)	(1.0206)	(1.0865)
Partner's Monthly Pay	0.000***	0.000	0.000***	-0.000	0.000***	-0.000	0.000***	-0.000
	(0.0000)	(0.0000)	(0.0000)	(0.0000)	(0.0000)	(0.0000)	(0.0000)	(0.0000)
Partner's Age	0.004	-0.005	0.004	-0.005	0.014	-0.074	-0.005	-0.082
	(0.0064)	(0.0047)	(0.0070)	(0.0048)	(0.0884)	(0.0954)	(0.0848)	(0.0905)
Partner's Age square	-0.005	0.016	-0.007	0.016	0.038	0.254	0.089	0.277
	(0.0164)	(0.0118)	(0.0180)	(0.0122)	(0.2217)	(0.2402)	(0.2115)	(0.2266)
Partner's Age cube	0.000	-0.000*	0.000	-0.000	-0.001	-0.002	-0.001	-0.003
	(0.0001)	(0.0001)	(0.0001)	(0.0001)	(0.0017)	(0.0019)	(0.0016)	(0.0018)
Partner's Experience	0.000	0.004*	-0.001	0.004	0.049	0.063	0.051	0.060
	(0.0029)	(0.0026)	(0.0032)	(0.0026)	(0.0462)	(0.0477)	(0.0447)	(0.0461)
Partner's Experience square	-0.004	-0.043*	0.008	-0.042*	-0.468	-0.702*	-0.514	-0.677*
	(0.0253)	(0.0232)	(0.0277)	(0.0233)	(0.3992)	(0.3957)	(0.3870)	(0.3853)
Partner's Experience cube	0.000	0.001**	-0.000	0.001*	0.010	0.017**	0.012	0.017**
	(0.0005)	(0.0005)	(0.0006)	(0.0005)	(0.0089)	(0.0085)	(0.0087)	(0.0083)
Partner Has a Degree	-0.010	0.004	-0.008	0.005	-0.149	-0.099	-0.165	-0.110
	(0.0110)	(0.0080)	(0.0120)	(0.0081)	(0.1596)	(0.1669)	(0.1597)	(0.1656)
Constant	-0.532***	0.239*	-1.037***		-10.767***	-18.038***	-9.868***	-17.016***
	(0.0877)	(0.1355)	(0.1113)		(0.8509)	(1.3845)	(0.8498)	(1.3792)
Time (joint significance)	8.76***	1.57	52.30***	10.73	22.94***	13.64*	15.60**	12.93*
Mundlak (joint significance)						336.36***		331.58***
N	27,674	27,674	27,674	24,722	27,674	27,674	27,674	27,674

Cluster robust standard errors are reported in parentheses; * $p < 0.1$, ** $p < 0.05$, *** $p < 0.01$
